# Alterations of voluntary behavior in the course of disease progress and pharmacotherapy in mice with collagen-induced arthritis

**DOI:** 10.1186/s13075-019-2071-z

**Published:** 2019-12-12

**Authors:** Yohsuke Oto, Yukari Takahashi, Daitaro Kurosaka, Fusao Kato

**Affiliations:** 1 Division of Rheumatology, Department of Internal Medicine, Jikei University School of Medicine, 3-25-8 Nishi-shimbashi, Minato-ku, Tokyo, Japan; 2 Department of Neuroscience, Jikei University School of Medicine, 3-25-8 Nishi-shimbashi, Minato-ku, Tokyo, Japan; 3 Center for Neuroscience of Pain, Jikei University School of Medicine, Minato-ku, Tokyo, Japan

**Keywords:** Rheumatoid arthritis, Collagen-induced arthritis, Arthritis-pain discordance, Wheel running, Temperature preference, Circadian rhythm, Tofacitinib, Astrocytes

## Abstract

**Background:**

Rheumatoid arthritis (RA) is an autoimmune disease characterized by chronic synovitis and bone destruction at the joints, causing pain and motor disturbance. Despite the better control of inflammation and joint deformity afforded by modern disease-modifying anti-rheumatic drugs, many patients with RA remain dissatisfied with their treatment, primarily because of sensory-emotional distress. Pre-clinical tests that can evaluate not only the symptoms of arthritis but also the associated pain as sensory-emotional experience are urgently needed.

**Methods:**

Here, we introduce two types of novel methods for evaluation of voluntary behavior in a commonly used model of RA (collagen-induced arthritis; CIA) in male mice. First, spontaneous motor activity was assessed with a running wheel placed in home cages and the number of rotations was continuously recorded in a 12:12-h light environment. Second, temperature preference was assessed by measuring the time spent in either of the floor plates with augmenting (25 to 49 °C) or fixed temperature (25 °C). We also evaluated the effects of tofacitinib on CIA-associated changes in voluntary wheel running and temperature preference.

**Results:**

We detected a significant decrease in voluntary wheel running, a significant shift in the distribution of movement in the dark phase, and a significant increase in the time spent in warmer environments than the room temperature in the mice with CIA. These alterations in voluntary behavior have never been described with conventional methods. We also revealed tofacitinib-resistant significant changes in the voluntary behavior and choice of temperature despite significant mitigation of the symptoms of arthritis.

**Conclusions:**

We described for the first time significant alterations of the voluntary behavior of the mice with CIA during the clinical periods, indicating that the overall physical/motivational states and its circadian variation, as well as the specific preference to a certain environmental temperature, are modified in the mice with CIA, as observed in human patients. Some of these did not parallel with the conventional arthritis scores, particularly during the pharmacotherapy suggesting that mice with CIA show not only the peripheral symptoms but also the central consequences. The use of these approaches would also help clarify the biological mechanisms underlying physician-patient discordance in the assessment of RA.

## Background

Rheumatoid arthritis (RA) is an autoimmune disease characterized by chronic synovitis and bone destruction at the joints. These pathological manifestations dramatically diminish the quality of life in patients with RA primarily as a result of motor disturbance as well as the pain associated with the disease. Recent advances in disease-modifying anti-rheumatic drugs, including the advent of biological agents and JAK inhibitors, enable now clinical remission even in previously intractable cases of RA. However, despite the better control of joint inflammation and deformity achieved by these drugs, many patients with RA remain subjectively dissatisfied with their treatment. For example, the subjective global assessment of the disease by the patient often does not match the assessment by the physician, particularly with respect to complaints of pain, which often persists despite improved joint symptoms [1–4]. To achieve higher remission and patient satisfaction rates, it is important to understand the mechanisms underlying the discordance between objective evaluation of symptoms and subjective assessment of pain in RA. To optimize the pharmacological treatments available for RA, methods are needed that can evaluate not only the symptoms of arthritis but also the pain and unpleasantness associated with experimental animal models of RA.

In most of the non-clinical studies of RA reported to date, withdrawal responses elicited by noxious mechanical stimuli have been used as a measure of pain. However, this approach has two significant limitations when used to evaluate pain-related symptoms in RA. First, the pain described by patients with RA often occurs at rest or during activities of daily living and not necessarily with noxious stimulation [5]. Therefore, pain-associated spontaneous behavior should be assessed in relation to voluntary movement and environmental sensory input. Second, the synovial inflammation and joint destruction associated with RA potentially interfere with reflexogenic rapid escape behavior, reducing the reliability and reproducibility of the measurements. As pain is an “unpleasant sensory and emotional experience” (the definition by the International Association for Study of Pain) and not a nociception [6], evaluation of pain in the animals with RA, not simple nociception-evoked reflexogenic responses, is necessary to advance our understanding of the neuronal mechanisms of central pain sensitization in RA models [7–11]. This would lead to development of novel therapies targeting both joint pathology and pain to improve patients’ quality of life.

Here, we propose a novel method for evaluation of voluntary behavior in a commonly used model of RA, the collagen-induced arthritis (CIA) in mice. In this model, we evaluated both changes in spontaneous locomotor activity and changes in preference for a particular ambient temperature in the course of development of and recovery from arthritis. We found a discordance between the objective signs of arthritis and voluntary behavior during treatment with a JAK inhibitor, tofacitinib, a clinically effective anti-rheumatic drug. This finding would help identify the biological mechanisms underlying the mismatch between assessment of RA activity by physicians and patients [1].

## Methods

### Experimental animals and ethical considerations

Male DBA/1 J mice (aged 5–7 weeks) purchased from Japan SLC (Shizuoka, Japan) were used in the study. The mice were housed in groups of 5–6 and maintained at a temperature of 22 ± 2 °C on a 12-h light/dark cycle (7 am to 7 pm), with food and water available ad libitum. The results of the wheel running test were recorded on a one-mouse-per-cage basis.

### Induction of arthritis

**Fig. 1:**
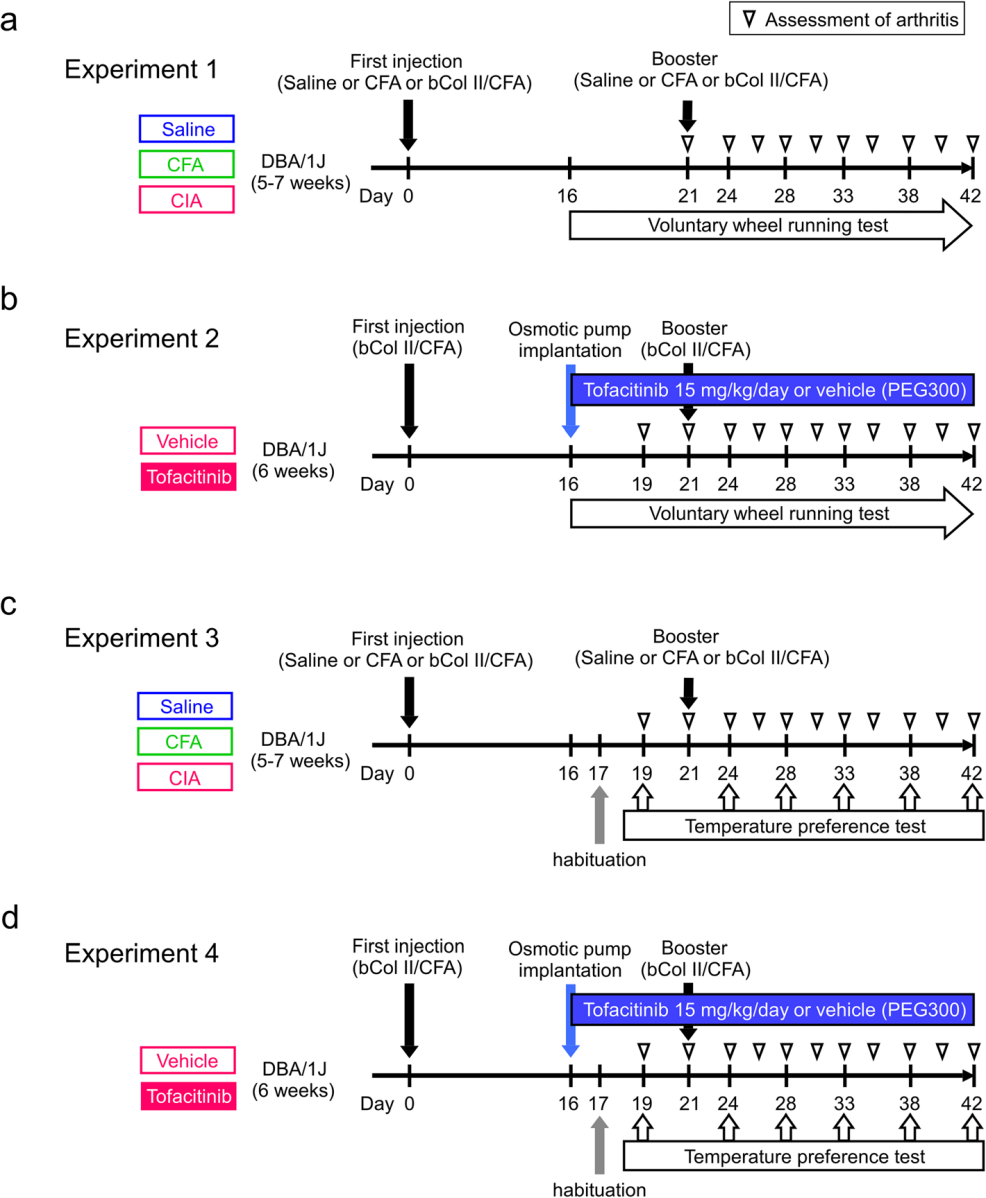
Experimental protocols. **a** Experiment 1. Collagen-induced arthritis (CIA) was induced in mice by a first injection of bovine type II collagen (bCol II) with complete Freund’s adjuvant (CFA) on day 0 and a booster injection on day 21 (black arrows). The control groups were injected with saline or CFA. The mice were single-housed in home cages with running wheels from day 16, and the number of wheel rotations was measured continuously (voluntary wheel running test). The arthritis score was checked on days 21, 24, 26, 28, 31, 33, 35, 38, 40, and 42 (open triangles). **b** Experiment 2. CIA was induced by a first injection of bovine type II collagen/CFA on day 0 and a booster injection on day 21. The CIA mice received tofacitinib 15 mg/kg/day or vehicle (PEG300) by subcutaneous osmotic pump infusion from day 16 (blue arrow). A voluntary wheel running test was performed from day 16 onwards. The arthritis score was assessed on days 19, 21, 24, 26, 28, 31, 33, 35, 38, 40, and 42 (open triangles). **c** Experiment 3. Each model was created in the same way as for experiment 1. The temperature preference test was performed on days 19, 24, 28, 33, 38, and 42 (open arrows) after habituation on day 17 (gray arrow). The arthritis score was assessed on days 19, 21, 24, 26, 28, 31, 33, 35, 38, 40, and 42 (open triangles). **d** Experiment 4. Induction of CIA and drug administration were performed in the same way as in experiment 2. The temperature preference test was performed on days 19, 24, 28, 33, 38, and 42 (open arrows) after habituation on day 17 (gray arrow). The arthritis score were assessed on days 19, 21, 24, 26, 28, 31, 33, 35, 38, 40, and 42 (open triangles)

At around 6–8 weeks of age, the mice were used to develop a model of CIA (Fig. 1). The immunization procedure consisted of two intradermal injections of (1) bovine type II collagen (Collagen Research Center, Tokyo, Japan; 200 μg/mouse) dissolved in 0.1 *M* acetic acid (4 mg/mL) emulsified with complete Freund’s adjuvant (2 mg/mL or 1 mg/mL; CFA; Becton Dickinson and Company, Franklin Lakes, NJ), (2) emulsified CFA without bovine type II collagen (2 mg/mL or 1 mg/mL), or (3) saline to the base of the tail under isoflurane anesthesia (3% in 100% O_2_) separated by 20 days (black arrows in Fig. 1). The CIA group received solution 1 with CFA 2 mg/mL on day 0 (the first injection day) and solution 1 with CFA 1 mg/mL as a booster injection on day 21. The CFA group received solution 2 (2 mg/mL) on day 0 and solution 2 with 1 mg/mL on day 21. The saline group received solution 3 (an equivalent volume of saline) on days 0 and 21. The CFA and saline groups served as controls for the CIA group. In a minority of the mice, CFA caused ulceration extending from the base of the tail to the anus (CIA group, 1 of 26; CFA group, 4 of 28; saline group, 0 of 28) on day 21. These mice were excluded from the following assessments.

### Experimental designs

Four types of experiments were performed in this study (Fig. 1a–d). Experiments 1 and 2 were voluntary wheel running tests, and experiments 3 and 4 were the temperature preference test. Mice were divided into 3 groups for experiments 1 and 3: a saline group (*n* = 10; 10 mice for the 2 experiments), a CFA group (CFA mice, *n* = 10; 10 mice for the 2 experiments), and a CIA group (CIA mice, *n* = 11; 9 mice for the 2 experiments; Fig. 1a, c). In experiments 2 and 4, the CIA mice were divided into a vehicle group (*n* = 8; 15 mice for experiments 2 and 4) and a tofacitinib group (*n* = 8; 14 mice for experiments 2 and 4) according to the drug administered (blue arrows in Fig. 1b, d). Additional experiments were performed to analyze hind paw thickness, body weight, and ankle joint histology, as well as mRNA expression of astrocyte (*Gfap*) and microglia (*Cd11b*) markers in the spinal cord (see sections below for individual methods). Eight mice were used in the saline group, 4 in the CFA group, and 5 in the CIA group. In total, 122 mice were used. Every effort was made to reduce the number of animals used and to minimize suffering.

### Assessment of arthritis

Clinical symptoms of arthritis were evaluated in each limb and scored as follows: 0, no evidence of erythema or swelling; 1, erythema and swelling confined to the digits, tarsals, or ankle joint; 2, erythema and swelling extending from the ankle to the tarsals; or 3, erythema and severe swelling extending from the ankle to the tarsals [12]. Scoring was performed by an experienced investigator every 2–3 days after day 19 or day 21 (open triangles in Fig. 1) holding 1 mouse at a time. This assessment was not fully blinded because the etiological grouping could not be hidden.

An arthritis score (0–12; the sum of scores for all 4 limbs) was recorded, and the incidence of arthritis (0–1) was defined as the ratio of non-zero-scored arthritic limbs to all 4 limbs. At every second arthritis score measurement (i.e., on days 19, 24, 28, 33, 38, and 42), mice were anesthetized with 3% isoflurane and the thickness of each hind paw was measured using an electronic caliper (AD5764; A&D Company, Tokyo, Japan). The mice were returned to the home cage immediately after recovery from anesthesia. Body weight was monitored throughout the study.

### Quantitative reverse transcription PCR

On day 45, the mice were perfused transcardially with ice-cold phosphate-buffered saline under deep anesthesia (pentobarbital sodium; 50 mg/kg, intraperitoneally) and the spinal cord was removed at L3–L5. Total RNA was extracted using an RNeasy Lipid Tissue Mini Kit (Qiagen, Tokyo, Japan) according to the manufacturer’s protocol. Real-time polymerase chain reaction (PCR) was performed using an Applied Biosystems StepOne Plus Real-Time PCR System (Thermo Fisher Scientific, Waltham, MA) using TaqMan probes and primers (Thermo Fisher Scientific) for *Gfap* (Mm01253033_m1), *Cd11b* (Mm00434455_m1), and *Gapdh* (Mm99999915_g1). Expression levels were normalized using *Gapdh* and analyzed by the ΔΔCT method [13]. mRNA expression in the spinal cord was represented as the value relative to the average value for the saline or vehicle group.

### Histology of ankle joint and spinal cord

On day 42, the mice were perfused transcardially with ice-cold phosphate-buffered saline followed by 4% paraformaldehyde in phosphate buffer under deep anesthesia (pentobarbital sodium; 50 mg/kg, administered intraperitoneally). The L4 spinal cord was removed, as well as the ankle joints. The L4 spinal cord was post-fixed in phosphate buffer containing 4% paraformaldehyde for 2 h at 4 °C, placed in 20% sucrose solution for 48 h at 4 °C, and frozen in optimal cutting temperature compound. Transverse sections of the spinal cord (20 μm thick) were cut using a cryostat and thaw-mounted onto glass slides. Sections were incubated in blocking solution (1% bovine serum albumin containing 0.3% Triton X-100 in phosphate-buffered saline) for 1 h at room temperature and then incubated for 24 h at 4 °C with mouse anti-mouse GFAP (1:1000, G3893, Sigma, St. Louis, MO) followed by incubation with Alexa Fluor 568-conjugated goat anti-mouse IgG (1:1000, Thermo Fisher Scientific, Rockford, IL) as the secondary antibody for 2 h at room temperature. Images were acquired using a laser confocal microscope (FV1200; Olympus, Tokyo, Japan). The ankle joints were post-fixed using 4% paraformaldehyde in phosphate buffer for 6 h, defatted in 100% ethanol, and decalcified for 5 days in 10% ethylenediaminetetraacetic acid solution at 4 °C; they were then embedded in paraffin and cut into 4-μm sections. The sections were deparaffinized and stained with hematoxylin and eosin according to standard procedures.

### Voluntary wheel running test (experiments 1 and 2)

We used 8 measurement cages with automatic-counting running wheels (SW-15, Melquest, Toyama, Japan). One mouse was single-housed in a measurement cage from days 16 to 42, and spontaneous wheel rotation was continuously recorded (CIF3Win, Melquest). Each home cage was separated by a wall and illuminated separately with LED lights (on a 12-h light-dark cycle; lights on at 7 am). On days 24, 31, and 38, between 11 am and 1 pm, the measurement chambers were replaced with cleaned ones containing fresh floor bedding, a full water bottle, and a full chow dispenser. Rotation measurement was halted during the replacement process, and the arthritis score of each mouse was assessed. This period is expressed as white (blank) time in the color scale display in Additional file 2: Figure S2. Mice allocated to the 3 groups in experiment 1 and those allocated to the 2 groups in experiment 2 were randomly assigned to each of the 8 cages. The 27-day session was repeated 4 and 2 times in experiments 1 and 2, respectively. The number of rotations during each 30-min interval was counted, and the time series were then analyzed using Excel and Igor Pro (WaveMetrics, Tigard, OR). The centroid time for the night-time rotation was calculated as follows:

centroid time = Σ(*r*(*t*)*t*)/Σ(*r*(*t*)) + 19:00, where *t* is the time in minutes from the beginning of the dark period (i.e., 7 pm) and *r*(*t*) is the rotation number at time *t*. This centroid time represents the distribution of the rotation during the observation time (i.e., when and how many rotations were observed).

### Temperature preference test (experiments 3 and 4)

The mice were placed on the middle of two adjacent hot plates (16.5 cm × 16.5 cm; BIO-T2CT, Bioseb, Vitrolles, France) and allowed to explore them freely. The temperature of each plate was controlled separately using the T2CT software supplied by the manufacturer. One of these plates had a continuously increasing temperature (plate 1) from the room temperature to higher temperature range (from 25 to 50 °C at a constant rate of 1 °C/min), and the other had a fixed temperature at room temperature (plate 2; 25 °C). The mice were set free to choose between two plates of different temperatures, none of which was not necessarily aversive, according to their preferred comfort. The movement of the mouse was captured using a USB camera fixed above the plates under red illumination and analyzed with the Bioseb video-tracking system. For habituation, the mice were placed on the hot plates at a fixed temperature (25 °C) for 25 min on day 17. A temperature preference test was performed on days 19, 24, 28, 33, 38, and 42. Based on the movement of the mice within the first 24-min observation period, a preference ratio, dwell time, and transition number were estimated as indices of temperature preference-dependent behavior as follows. The preference ratio was the total amount of time spend on plate 1 relative to the total observation period. This ratio is an indicator of the extent to which the mice preferred the plate with increasing temperature at each time point. The dwell time was the mean duration spent continuously on plate 1 within every 2-min time frame (Additional file 5: Figure S5A) and every 6-min time frame (Additional file 5: Figure S5B). The transition number was the number of times the mice moved from plate 1 to plate 2 and vice versa. The trajectory of the spontaneous displacement of the mice in Additional file 4: Figure S4 was analyzed semi-automatically with tracking software (ANY-maze, Stoelting Co., Wood Dale, IL).

### Treatment of CIA with tofacitinib

Tofacitinib, a JAK inhibitor, was purchased from Selleck Chemicals (S2789, Houston, TX). Mice were randomly assigned to receive either tofacitinib in poly (ethylene glycol) (PEG)300 (100 mg/mL; Nacalai Tesque, Kyoto, Japan) at 15 mg/kg/day or vehicle alone (PEG300) by osmotic pump infusion (Alzet Model 2004, 0.25 mL/h, 28 days; Durect, Cupertino, CA) [14]. On day 16, each mouse was anesthetized with isoflurane 3% and its dorsal surface was shaved. A 1-cm incision was made on the back of the mouse, and the pump was inserted subcutaneously with the ejection hole directed caudally [15]. It should be noted that this administration protocol of tofacitinib starting on day 16 is distinct from that in clinical cases. In RA patients, the administration of anti-rheumatism drug cannot be started before the medical diagnosis of RA. The incision site was sutured with a wound clip. After experiment 2 and experiment 4, the osmotic pump was taken out to confirm appropriate reduction in solution volume after approximately 30 days of administration and that there was no damage to the pumps.

### Statistical analysis

All statistical analyses were performed using R (The R Foundation for Statistical Computing, Vienna, Austria, version 3.41) or EZR (Saitama Medical Center, Jichi Medical University, Saitama, Japan). EZR is a graphic user interface for R [16]. More precisely, it is a modified version of R commander (version 1.36) and is designed for easier access to statistical functions frequently used in biostatistics. The three groups were compared by one-way analysis of variance (ANOVA) followed by Tukey’s post hoc test. When heteroscedasticity was confirmed by Bartlett’s test, the Kruskal-Wallis test followed by the Steel-Dwass post hoc multiple comparison test was used instead. *F* values with the degrees of freedom and *p* values of the ANOVA test are described in the main text of the “Results” section, and the *p* values and type of the post hoc tests are shown with “*” or “#” marks in the figures and the legends, respectively. Friedman’s test or one-way repeated-measures ANOVA was used to analyze the time-dependent changes in each group. Two-way repeated-measures ANOVA was used to compare time-dependent changes between two groups. When the interaction effects were significant, the Student’s *t* test or Welch’s *t* test were used for the comparisons of the data at each point. The paired *t* test was used to compare paired groups. The Mann-Whitney *U* test was used to compare the mean ranks of two groups. Correlation analysis was performed using Kendall’s correlation. Statistical significance of differences in the rank correlation coefficient was assessed using the *Z*-transform method after translating Kendall’s tau to *r* [17]. Differences were considered to be significant when the *p* value was < 0.05.

## Results

### Three symptomatic phases of CIA in mice

The arthritis score was analyzed in all mice used in this study. In all the experiments, manifestations of arthritis were observed only in the CIA group (in 24 of 25 mice; Additional file 1: Figure S1A). The first signs of arthritis (i.e., arthritis in at least one limb) were not observed until day 26. Arthritis-related symptoms in the CIA group were accompanied by changes in hind paw thickness, body weight, and ankle joint pathology (Additional file 1: Figure S1B–S1D; these figures contain data from distinct experimental protocols, but the CIA, CFA, and saline groups were always clearly distinguished). These changes were not observed in the CFA group or saline group. We divided the observation period into the following three phases according to the time course of the changes in arthritic state: pre-clinical (days 18–25; a period with no detectable signs of arthritis), early clinical (days 26–34; a period with increasing scores and incidence), and late clinical (days 35–42; a period with sustained high scores and greater incidence; Additional file 1: Figure S1A). At the end of the late clinical period, there was infiltration of inflammatory cells and typical destruction of the cartilage in the ankle joint in the CIA group but not in the saline or CFA group (Additional file 1: Figure S1D). In a similar model of CIA, Inglis et al. have demonstrated that an increase in reactive astrocytes, but not microglia, in the lumber spinal dorsal horn characterizes the progress of arthritis, suggesting that peripheral arthritis also affects the central nervous system, which might have various neuronal consequences [7]. We examined whether our CIA also affects the glial cell expression in the central nervous system. On days 42–45, there was an increase in the number of reactive astrocytes in the dorsal horn (Additional file 1: Figure S1E), and a significant increase in expression of *Gfap* (*F*(2, 26) = 15.29, *p* < 0.01; one-way ANOVA) but not in that of *Cd11b* (*F*(2, 26) = 1.906, *p* = 0.169; one-way ANOVA) was also confirmed in the CIA group but not in the saline group or the CFA group (Additional file 1: Figure S1F). These results indicate that the symptoms of the joint inflammation and the alterations in the spinal cord glial cells in the clinical periods are characteristics of the mice with CIA.

### Effects of arthritis on voluntary wheel running (experiment 1)

**Fig. 2:**
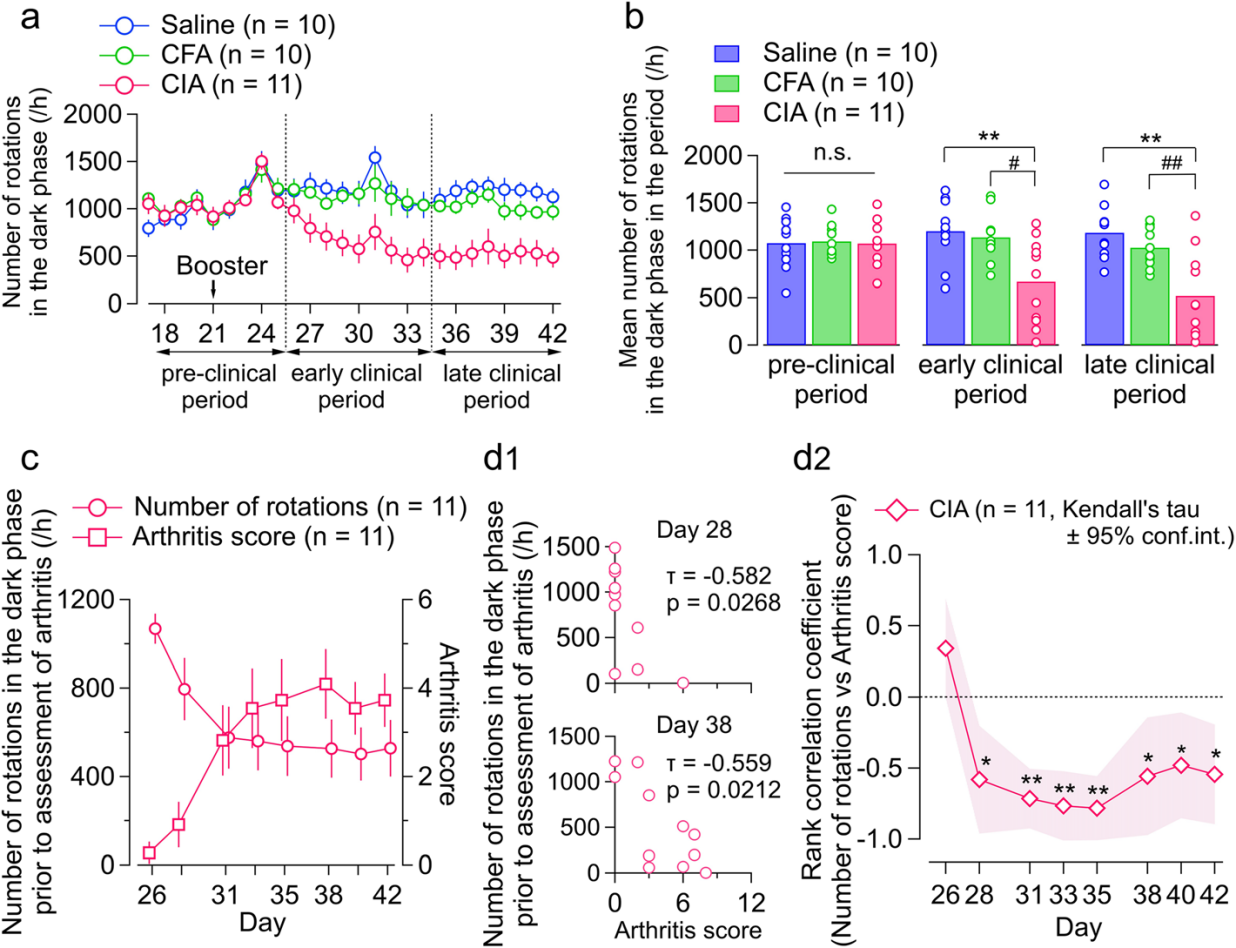
Effects of arthritis on voluntary wheel running (experiment 1). **a** Time course of number of rotations per hour in the dark phase in the saline (blue), CFA (green), and CIA (red) groups. The values are shown as the mean ± standard error of the mean. **b** Mean number of rotations per hour in the dark phase during the pre-clinical period (days 18–25), early clinical period (days 26–34), and late clinical period (days 35–42). The bars indicate the average number of rotations, and each open circle indicates a value from a mouse. *p < 0.05, **p < 0.01, saline vs. CIA; ^#^*p* < 0.05, ^##^*p* < 0.01, CFA vs. CIA, by one-way ANOVA followed by Tukey’s test. **c** Time course of arthritis score (right y axis) and number of rotations per hour in the dark phase prior to assessment of arthritis (left y axis). Abscissa, days after first injection. The values are shown as the mean ± standard error of the mean. **d**_**1**_ Scatter plots showing the relationship between number of rotations in the dark phase prior to assessment of arthritis (*y* axis) and the arthritis score (*x* axis) on day 28 (above) and day 38 (below). The insets show the values for Kendall’s tau and the *p* value for zero correlation **d**_**2**_ Time course of rank correlation coefficient in the CIA group. The hashed zone indicates the 95% confidence interval for Kendall’s tau. *p < 0.05, **p < 0.01, by Kendall’s correlation analysis. CFA, complete Freund’s adjuvant; CIA, collagen-induced arthritis; n.s., not significant

Using this RA model, we continuously evaluated the voluntary movements of the mice from days 16 to 42 to analyze how the development of arthritis affects spontaneous behavior. The panels in Additional file 2: Figure S2 are representative examples of the time course for number of rotations and arthritis scores in single mice from the saline, CFA, and CIA groups displayed in respective color scales. As expected, most wheel running behavior was observed in the dark phase regardless of group allocation in the pre-clinical period (Additional file 2: Figure S2 and Additional file 3: Figure S3A). In the early and late clinical periods, the CIA group, but not the saline group or CFA group, showed a marked decrease in the number of rotations in the dark phase. This decrease coincided with an increase in the arthritis score for the fore and hind paws in the CIA group (right side of each panel in Additional file 2: Figure S2; the arthritis score). The CFA and saline groups did not show any increase in the arthritis score (Additional file 3: Figure S3B). Figure 2a provides a summary of the changes in number of rotations in the dark phase during the pre-clinical and clinical periods. The average number of rotations in the CIA group, but not in the saline group or CFA group, decreased gradually after the appearance of arthritis symptoms on approximately day 26 and remained smaller during the observation period (until day 42). In the CIA group, the mean number of rotations in the dark phase was significantly smaller than that in the other two groups in the early (*F*(2, 28) = 6.973, *p* < 0.01; one-way ANOVA) and late (*F*(2, 28) = 11.75, *p* < 0.01; one-way ANOVA) clinical periods but not in the pre-clinical period (*F*(2, 28) = 0.027, *p* = 0.973; one-way ANOVA; Fig. 2b).

Interestingly, this decrease in the number of rotations in the CIA group was accompanied by a shift in distribution of the number of rotations during the night (histograms at the bottom of Additional file 2: Figure S2). For example, in the representative CIA mouse shown in Additional file 2: Figure S2, the number of rotations in the late-dark phase decreased more than that in the early-dark phase from days 23 to 34. This resulted in a shift of centroid time to an earlier time in the night (pink and red arrowheads in Additional file 2: Figure S2, CIA bottom), suggesting that the decrease in locomotor activity was more pronounced in the late-dark phase. This shift in the distribution of locomotor activity toward an earlier time (i.e., earlier shift of centroid time) became more manifest in the course of development of arthritis; the centroid time became significantly earlier in the CIA group than in the other study groups in both the early (*F*(2, 28) = 5.547, *p* < 0.01; one-way ANOVA) and late (*F*(2, 28) = 5.672, *p* < 0.01; one-way ANOVA) clinical periods (Additional file 3: Figure S3C, D).

We then sought to determine whether or not the decrease in the number of rotations was related to the symptoms of arthritis. Figure 2c shows the time course of the number of rotations and that of the arthritis score on the same time axis, indicating that each of these is a mirror image of the other. To test if a mouse with a higher arthritis score had a greater decrease in the number of rotations in the dark phase, we estimated the rank-order correlation between these values measured in each mouse (Fig. 2d). We found that they were negatively and significantly correlated from day 28 to day 42 (*τ* = − 0.582, *p* < 0.05 [day 28]; *τ* = − 0.716, *p* < 0.01 [day 31]; *τ* = − 0.767, *p* < 0.01 [day 33]; *τ* = − 0.784, *p* < 0.01 [day 35]; *τ* = − 0.559, *p* < 0.05 [day 38]; *τ* = − 0.482, *p* < 0.05 [day 40]; *τ* = − 0.545, *p* < 0.05 [day 42]; Kendall’s correlation analysis). This correlation was not observed in the CFA group and the saline group because the arthritis score was consistently zero in these groups (Additional file 3: Figure S3B).

### Influence of tofacitinib on CIA-associated change in voluntary wheel running (experiment 2)

**Fig. 3:**
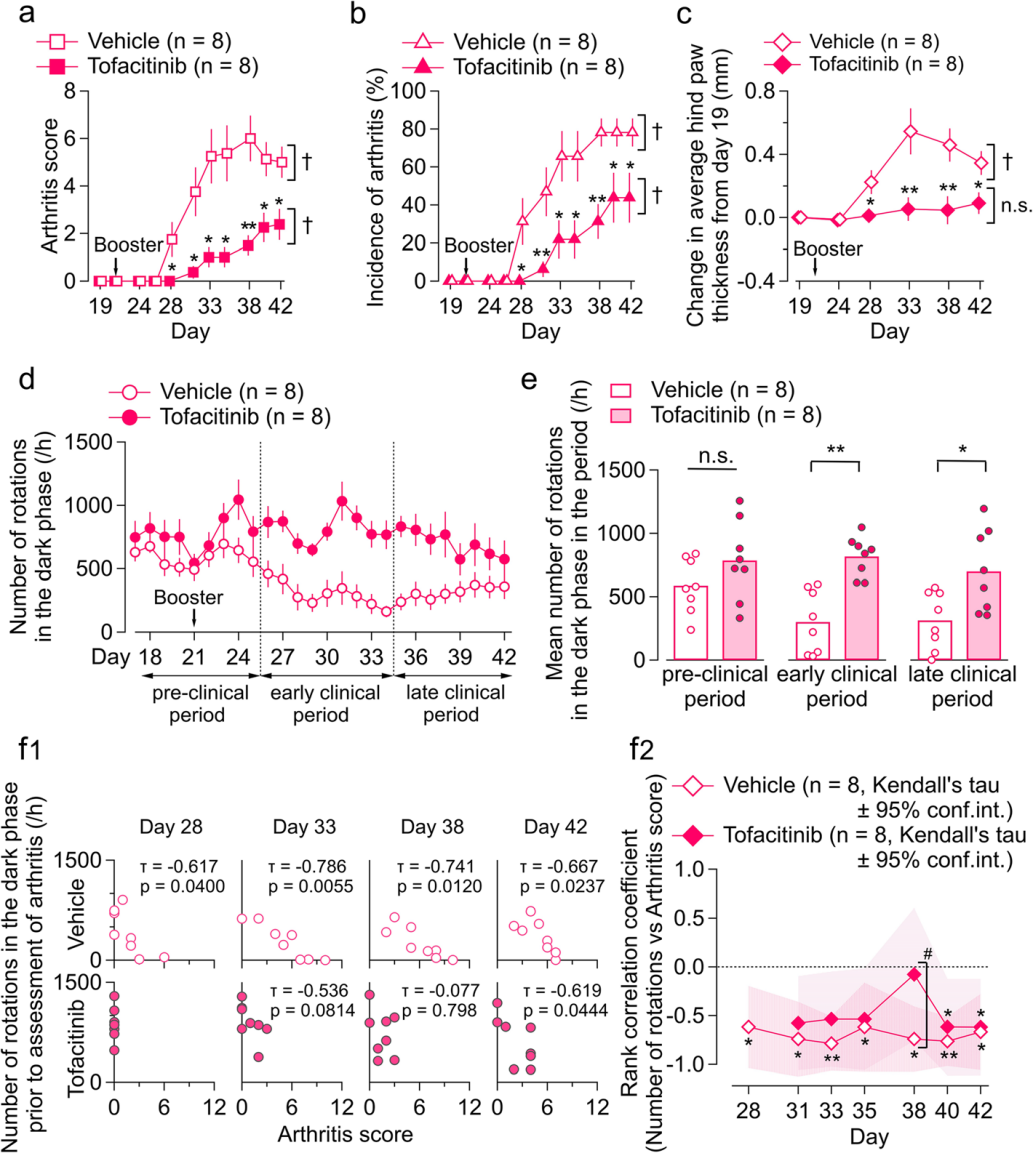
Influence of tofacitinib on CIA-associated change in voluntary wheel running (experiment 2). **a**–**c** Time course of arthritis score (**a**), incidence of arthritis (**b**), and change in average hind paw thickness from day 19 (**c**) in tofacitinib-treated and vehicle-treated CIA mice. Abscissa, days after first injection. The values are shown as the mean ± standard error of the mean. The interaction effects were significant (*p* < 0.05) by two-way repeated-measures ANOVA in **b** and **c**. *p < 0.05, **p < 0.01, tofacitinib vs. vehicle; by the Mann-Whitney *U* test (**a**) and Student’s *t* test (**b**, **c**). ^†^*p* < 0.01; by Friedman’s test (**a**) and one-way repeated-measures ANOVA (**b**, **c**). **d** Time course of number of rotations per hour in the dark phase in tofacitinib-treated (filled circle) and vehicle-treated (open circle) CIA mice. The values are shown as the mean ± standard error of the mean. **e** Mean number of rotations in the dark phase in the pre-clinical, early clinical, and late clinical periods in tofacitinib-treated and vehicle-treated CIA mice. The bars indicate the average number of rotations, and each circle indicates the value for one mouse. *p < 0.05, **p < 0.01, tofacitinib vs. vehicle; Student’s *t* test. **f**_**1**_ Scatter plots showing the relationship between number of rotations in the dark phase prior to the assessment of arthritis (*y* axis) and arthritis scores (*x* axis) in the tofacitinib-treated (below) and vehicle-treated (above) CIA mice on days 28, 33, 38, and 42. The insets show the values for Kendall’s tau and the *p* value for zero correlation. **f**_**2**_ Time course for rank correlation coefficient between number of rotations and the arthritis score in tofacitinib-treated and vehicle-treated CIA mice. The hashed zone indicates the 95% confidence interval of Kendall’s tau. *p < 0.05, **p < 0.01, by Kendall’s correlation analysis. ^#^*p* < 0.05, by *Z*-transform method. CIA, collagen-induced arthritis; n.s., not significant

Next, we sought to determine how the widely used anti-rheumatic drug tofacitinib affects CIA-associated inhibition of voluntary wheel running. CIA mice received tofacitinib or vehicle (PEG300) at a dose of 15 mg/kg/day by subcutaneous osmotic pump infusion from day 16. Tofacitinib lowered the mean arthritis score (Fig. 3a) and incidence of arthritis (Fig. 3b) after day 28 in mice with CIA. The hind paw thickness significantly increased during the course of arthritis progress in the vehicle group (*p* < 0.01), while it was not significantly changed in the tofacitinib group (*p* = 0.40; one-way repeated measures ANOVA with the Greenhouse-Geisser correction; Fig. 3c). The hind paw thickness differed between tofacitinib and vehicle groups at each day after day 28 (Fig. 3c). These results were in agreement with a previous report describing the effects of tofacitinib in a similar CIA model [14]. After day 26, the number of rotations decreased in the CIA mice receiving the vehicle, while those continuously treated with tofacitinib did not show this decrease (Fig. 3d). The number of rotations in the CIA mice treated with tofacitinib was significantly greater in the early (*t* (14) = − 5.002, *p* < 0.01; Student’s *t* test) and late (*t* (14) = − 2.753, *p* < 0.05; Student’s *t* test) clinical periods than in those treated with the vehicle (Fig. 3e). No significant difference between the CIA mice treated with tofacitinib and their untreated counterparts was observed in the pre-clinical period (*t* (14) = − 1.466, *p* = 0.165, not statistically significant, Student’s *t* test). In the early clinical period, the centroid time differed between the CIA mice that were and were not treated with tofacitinib (*t* (14) = − 2.973, *p* < 0.05; Student’s *t* test; Additional file 3: Figure S3E, F).

The negative correlation between the number of rotations and arthritis scores after day 28 in CIA mice as shown above (Fig. 2d) was also confirmed in CIA mice treated with the vehicle (Fig. 3f). However, treatment with tofacitinib disrupted this significant correlation on day 31 (tofacitinib, *τ* = − 0.741, vs. vehicle, *τ* = − 0.577), day 33 (*τ* = − 0.786 vs. *τ* = − 0.536), day 35 (*τ* = − 0.618 vs. *τ* = − 0.536), and day 38 (*τ* = − 0.741 vs. *τ* = − 0.077; Fig. 3f). Notably, the arthritis-rotation correlation was significantly smaller in the tofacitinib group than in the vehicle group on day 38 (*z* = − 2.3, *p* < 0.05; *Z*-transform method; Fig. 3f).

### Effects of arthritis on temperature preference (experiment 3)

Mice with CIA show shortened response latency to noxious heat radiation of fixed intensity to the hind paw [7, 18] and shortened latency to show licking of the hind paw or jumping behavior after placement on a hot plate of 52 °C [19]. The authors of these studies attributed these changes to inflammatory changes in the nociceptive neurons. However, these tests were made using noxious stimulation and would not suggest that the wide-range thermal sensation, which is maintained by combined activation of thermo-sensitive channels in TRP channel family expressed in DRG, is affected in RA animals. Instead of evaluating these “evoked” responses to single noxious thermal stimulations, we tried to evaluate the changes in spontaneous behavior in response to non-noxious environmental temperature that both animals and humans experience in daily life. For this purpose, we monitored the spontaneous behavior of the CIA mice when placed in an environment where they can choose between a compartment with a floor at room temperature and a compartment with a floor that has a higher temperature. Additional file 4: Figure S4 shows the representative patterns of the movements of the mouse from saline, CFA, and CIA groups in 24-min observation period in this chamber on day 38.

**Fig. 4:**
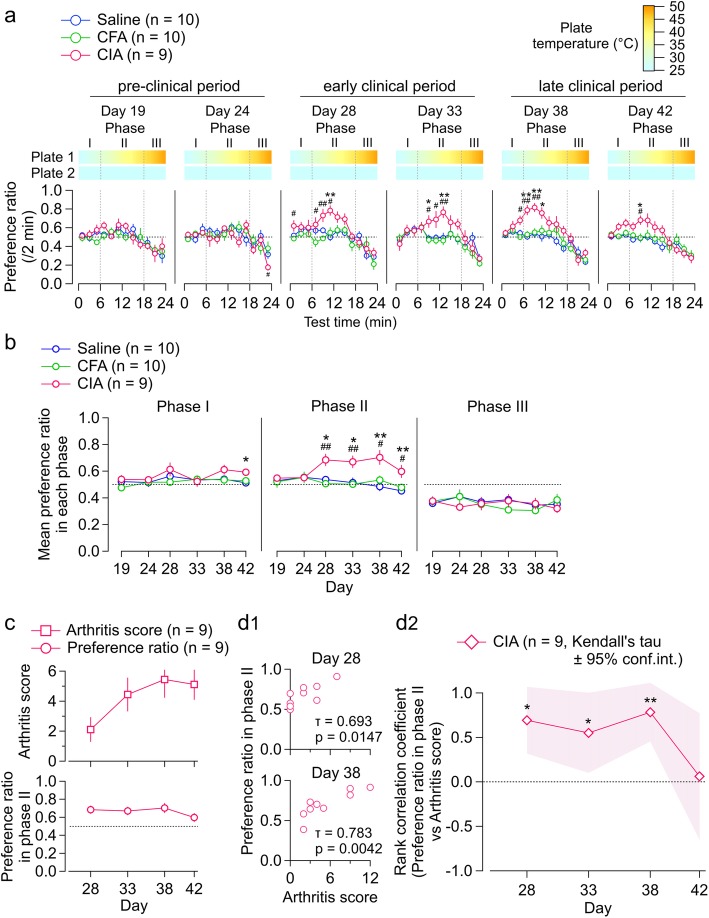
Effects of arthritis on temperature preference (experiment 3). **a** Test time-dependent changes in the preference ratio between two plates with fixed temperature (25 °C) and increasing temperature (1 °C/min from 25 °C for 24 min) in the pre-clinical period (days 19–24), early clinical period (days 28–33), and late clinical period (days 38–42) in the saline, CFA, and CIA groups. Ordinate, preference ratio per 2 min; abscissa, test time. The color scale above indicates the temperature of the plate at each time point. The test interval was divided in three phases as follows: 0–6 min, phase I; 6–18 min, phase II; and 18–24 min, phase III. The horizontal dotted line indicates a preference ratio of 0.5. **b** Time course of the mean preference ratio in each phase in the saline, CFA, and CIA groups. The horizontal dotted line indicates a preference ratio of 0.5. The values in **a** and **b** are shown as the mean ± standard error of the mean. *p < 0.05, **p < 0.01, saline vs. CIA; ^#^*p* < 0.05, ^##^*p* < 0.01, CFA vs. CIA, by one-way ANOVA followed by Tukey’s test. **c** Time course of arthritis score (upper *y* axis) and preference ratio in phase II (lower *y* axis) in the CIA group. **d**_**1**_ Scatter plots showing the relationship between the preference ratio in phase II (*y* axis) and arthritis score (*x* axis) on days 28 (above) and 38 (below). The insets show the values for Kendall’s tau and the *p* value for zero correlation. **d**_**2**_ Time course of rank correlation coefficient for the relationship between the preference ratio in phase II and the arthritis score in the CIA group. The hashed zone indicates the 95% confidence interval for Kendall’s tau. *p < 0.05, **p < 0.01, by Kendall’s correlation analysis. ANOVA, analysis of variance; CFA, complete Freund’s adjuvant; CIA, collagen-induced arthritis

Figure 4 shows the results of this temperature preference test. In the pre-clinical period, there was no significant difference in temperature change-dependent plate preference between the saline, CFA, and CIA groups (Fig. 4a; days 19 and 24). In contrast, in the early and late clinical periods, only the CIA group showed a distinct pattern of temperature preference; the preference ratio became significantly stronger (i.e., the mouse preferred to stay on the warmer plate) and mostly in the phase when the temperature difference was 6–18 °C (Fig. 4a, days 28–42). We divided the observation time of 24 min to three phases (I, II, and III) and analyzed them separately (Fig. 4b): phase І (0–6 min; plate 1 temperature, 25–31 °C), phase II (6–18 min; 31–43 °C), and phase ІІІ (18–24 min; 43 °C–49 °C). In phase II, the CIA group showed a distinct temperature preference when compared to the saline group and the CFA group but not in phases I and III after day 28 (i.e., only in the early and late clinical periods; Fig. 4b). More detailed analyses of the location of the mice revealed that the preference to stay on the warmer plate in the CIA group during the clinical period was accompanied by a prolonged dwell time and a reduced transition number (Additional file 5: Figure S5A). In the early-to-late clinical periods, the dwell time and transition number in the CIA group differed significantly from the values in the other groups (phase I in Additional file 5: Figure S5B) despite the lack of significant difference in the preference ratio in this phase. A plausible interpretation for this is that the CIA group showed reduced mobility for moving to/from plates, probably resulting from arthritis despite the similar temperature preference to saline and CFA groups (Fig. 4b, phase I). Overall, the preference for the 31–43 °C plate to the 25 °C plate is characteristic of mice with CIA in the early and late clinical periods. The preference ratio in phase II was significantly correlated with the arthritis score on day 28 (*τ* = 0.693, *p* < 0.05), day 33 (*τ* = 0.551, *p* < 0.05), and day 38 (*τ* = 0.783, *p* < 0.01; Kendall’s correlation analysis; Fig. 4c, d). The data for the phase II preference ratio in Fig. 4c are the same as those for phase II in Fig. 4b, but are shown again for ease of comparison with the arthritis score.

### Influence of tofacitinib on CIA-associated changes in temperature preference (experiment 4)

**Fig. 5:**
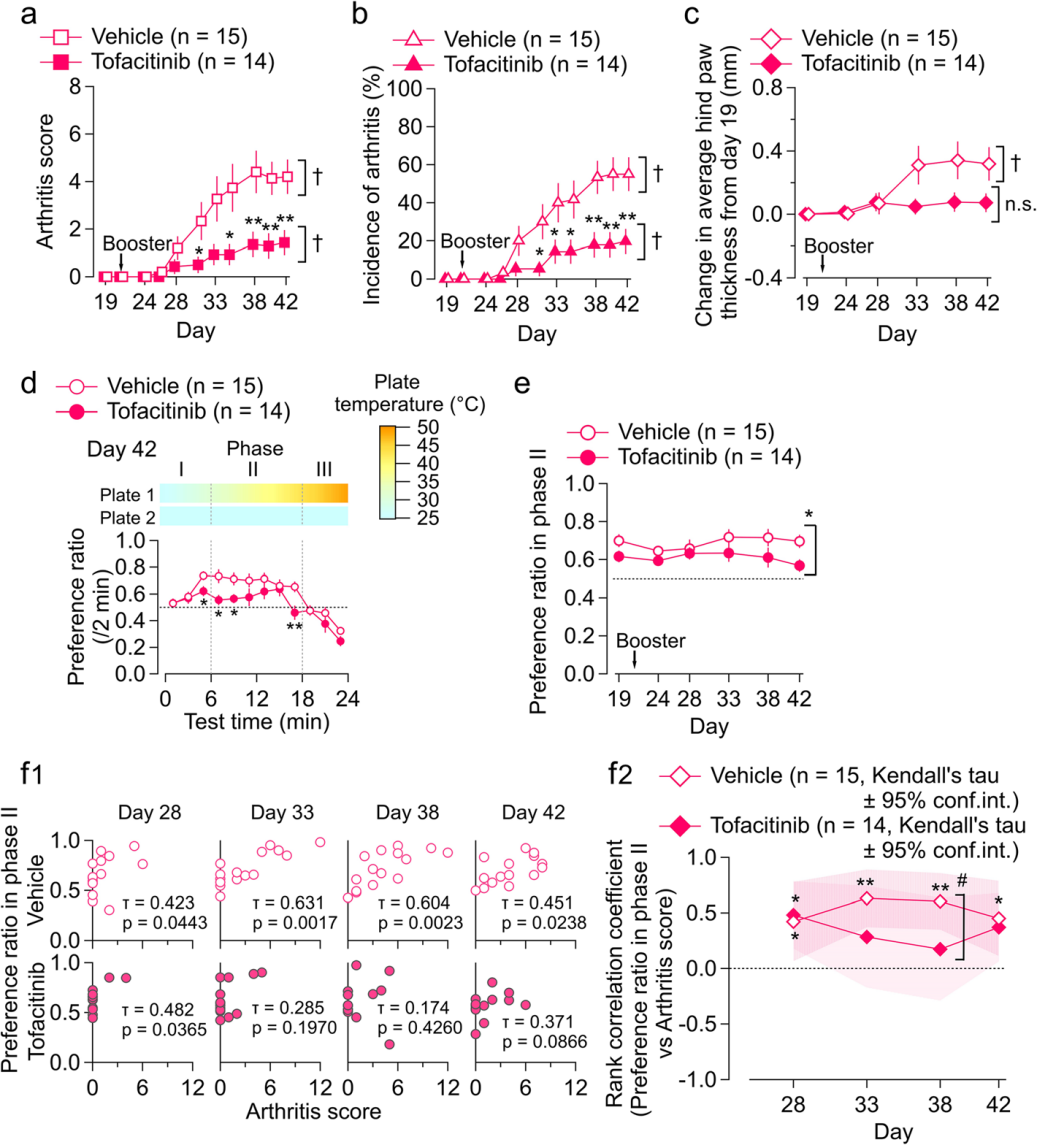
Influence of tofacitinib on CIA-associated changes in temperature preference (experiment 4). **a**–**c** Time course of arthritis score (**a**), incidence of arthritis (**b**), and change in average hind paw thickness from day 19 (**c**) in tofacitinib-treated and vehicle-treated mice with CIA. The values are shown as the mean ± standard error of the mean. The interaction effects were significant (*p* < 0.05) by two-way repeated-measures ANOVA in **b** and **c**. *p < 0.05, **p < 0.01, tofacitinib vs. vehicle; by the Mann-Whitney *U* test (**a**), Student’s *t* test (**b**), and Welch’s *t* test (**c**). ^†^*p* < 0.01; by Friedman’s test (**a**) and one-way repeated-measures ANOVA (**b**, **c**). **d** Time-dependent change in preference ratio on day 42 in the tofacitinib-treated (filled circle) and vehicle-treated (open circle) CIA mice. The ordinate indicates the preference ratio per 2 min. The color scale above indicates the temperature of each plate at each time point. The horizontal dotted line indicates a preference ratio of 0.5. The values are shown as the mean ± standard error of the mean. *p < 0.05, **p < 0.01, tofacitinib vs. vehicle; Student’s *t* test. **e** Time course of preference ratio for phase II (mean 6–18 min) in tofacitinib-treated and vehicle-treated CIA mice. The values are shown as the mean ± standard error of the mean. *p < 0.05, tofacitinib vs. vehicle; by two-way repeated-measures ANOVA. **f**_**1**_ Scatter plots showing the relationship between the preference ratio in phase II (*y* axis) and the arthritis score (x axis) for tofacitinib-treated (below) and vehicle-treated (above) CIA mice. **f**_**2**_ The time course of the rank correlation coefficient between the preference ratio in phase II and the arthritis score in tofacitinib-treated (filled diamond) and vehicle-treated (open diamond) CIA mice. The hashed zone indicates the 95% confidence interval for Kendall’s tau. *p < 0.05, **p < 0.01, by Kendall’s correlation analysis. ^#^*p* < 0.05, by the *Z*-transform method. CIA, collagen-induced arthritis; n.s., not significant

As described above, in the course of recovery during treatment with tofacitinib, there was a discordance between CIA-associated inhibition of voluntary wheel running and the arthritis score (day 38, Fig. 3f_2_). We examined whether or not such discordance also occurs with the temperature preference test. Tofacitinib significantly lowered the arthritis score (Fig. 5a) and the incidence of arthritis (Fig. 5b) in the cohort of mice that were used for this test. The hind paw thickness was significantly increased during the course of arthritis progress in the vehicle group (*p* < 0.01), while it was not significantly changed in the tofacitinib group (*p* = 0.36; one-way repeated measures ANOVA with the Greenhouse-Geisser correction; Fig. 5c), indicating that time-dependent paw thickening in the arthritis animals was attenuated in mice with tofacitinib treatments. Unlike the results in Fig. 3c for the mice with voluntary wheel running, we failed to find a significant between-group difference at any of the measurement days. These different results would be partly attributed to the differences in the home cage environment, such as the one-mouse-per-cage density and the continuous presence of the running wheel. The influences of these factors on the drug effects are subjects of future studies.

Figure 5d shows the time-dependent change in the preference ratio during the temperature rise on one of the plates on day 42. While the preference ratio in the vehicle-treated groups increased in phase II, it was not apparent in the tofacitinib-treated group. There was a significant difference in the preference ratio at 4–10 min and 16–18 min between the vehicle-treated and tofacitinib-treated groups (Fig. 5d) that was accompanied by a shorter dwell time and an increased transition number in the tofacitinib-treated group in phase II (Additional file 5: Figure S5C). Two-way repeated-measures ANOVA indicated significant effects of tofacitinib (*F*(1, 28) = 5.45, *p* < 0.05) with no significant interaction effect (Fig. 5e). The positive correlation between the phase II preference ratio and arthritis score after days 28–38 in CIA mice (as shown above in Fig. 4d) was also confirmed in CIA mice that received the vehicle after day 28 (Fig. 5f). However, treatment with tofacitinib disturbed this significant correlation on day 33 (vehicle, *τ* = 0.631, vs. tofacitinib, *τ* = 0.285), day 38 (*τ* = 0.604 vs. *τ* = 0.174), and day 42 (*τ* = 0.451 vs. *τ* = 0.371; Fig. 5f). The arthritis-preference ratio correlation was significantly smaller in the tofacitinib-treated group than in the vehicle-treated group on day 38 (*z* = 2.05, *p* < 0.05, *Z*-transform method; Fig. 5f_2_), as observed for the correlation of arthritis with number of rotations described above (Fig. 3f_2_).

## Discussion

In this study, we compared the symptoms of arthritis, voluntary locomotor activity, and preference for a particular environmental temperature between mice allocated to a saline group, a CFA group, and a CIA group and found the following. (1) The locomotor activity was significantly decreased, particularly in the latter half of he active (dark) period, after day 26 (5 days after the booster injection) and significantly correlated with the arthritis score after day 28 in the CIA group, but not the saline group or the CFA group. (2) The CIA group showed a significantly greater preference for floor temperatures in the middle range (31–43 °C) after day 28, whereas the saline and CFA groups did not. This tendency for mice with CIA to prefer middle range temperatures was significantly correlated with the arthritis score on days 28–38. (3) Sustained treatment with tofacitinib starting at day 16 significantly improved the arthritis score, increased the locomotor activity, and attenuated the middle range temperature preference, but their correlation with the arthritis score was disturbed around day 38. (4) The CIA group, but not the saline or CFA group, showed increased immunoreactivity of GFAP in the spinal dorsal horn and its mRNA level without a detectable change in *Cd11b* mRNA in the spinal cord at L3–L5. Altogether, these results indicate that evaluation of voluntary behavior using these methods is a reliable and promising approach to separately quantify the pathology of arthritis and pain-associated behavior. Plausible interpretations of the behavioral results are discussed below.

### Voluntary wheel running test

This study is the first to analyze the continuous changes in spontaneous locomotor activity in an RA model over a long period. This approach allows purely objective comparisons of daily changes in distance traveled per hour in the course of progression of arthritis or medication-induced improvement of the symptoms of arthritis without intermittently exposing the animals to particular environments. Such lengthy observation is necessary for evaluating the chronic course of rheumatic disease and its recovery with drug treatment. The overall decrease in spontaneous locomotor activity during continuous observation for more than 3 weeks in this study is in agreement with previous studies in which locomotor activity was sampled by monitoring movements in home cages for a shorter period in rodent or murine models of RA [7, 20–22]. The most straightforward interpretation is that nociception at the affected joint disturbs the movement of the limb necessary for rotating the running wheel. However, such a decrease in the number of rotations has been reported also in animal models of migraine [23], experimental autoimmune encephalomyelitis [24], and CFA-induced inflammatory pain in the hind paw [25] that do not necessarily show pathological changes in the joints. Indeed, changes in the number of rotations were not necessarily correlated with the arthritis score during treatment with tofacitinib in this study. This finding suggests that not only joint symptoms but also other factors, including a subjective sense of unpleasantness, fatigue, and widespread sensitization [5], might underlie this change. In particular, the early-clinical period, in which the significant reduction in the number of rotation in wheel running test was observed in this study, roughly corresponds to the period in which Inglis et al. have observed a significant increase in mechanical and thermal sensitivity in the very similar CIA model [7]. The authors of this article argued that in this augmented nociceptive behavior in the ~ 10-day post-arthritis period would have resulted from increased painful sensation rather than disabled joint function, which becomes more manifest in advanced stages after 10 days (i.e., after day 42 in our observation). The present results indicate that in this period before day 42 is also characterized by altered voluntary behavior in the situations with spontaneous non-forced running and with temperature-dependent choice of environment, which is not necessarily painful. As such, the present findings provide evidence that not only the tactile/thermal sensitivity in the periphery but also the brain-dependent behavioral choices in the spontaneous behavior are affected in the course of disease progress as well as pharmacotherapy in the mice with CIA. Interestingly, we found for the first time a correlation between the decrease in the number of rotations and the increase in the severity of arthritis in the early and late clinical periods (Fig. 2d). Moreover, continuous evaluation of spontaneous behavior enabled discordance between the arthritis score and behavior in the course of anti-rheumatic treatment (Fig. 3f) to be revealed for the first time. Such an approach may help identify the biological mechanisms underlying the discordance between subjective patient reports and objective evaluations by physicians.

Another advantage of the present approach is that the continuous running wheel assessment can also evaluate the intraday variation of the animal behavior. Symptoms of arthritis in RA show clear circadian variation in humans [26–28], and this advantage would be significant in translating findings in animal to human patients. Interestingly, in addition to the decrease in number of rotations during the dark phase, the CIA mice ran less in the later period of the dark phase as arthritis worsened (Additional file 3: Figure S3C, D). The mechanism underlying this shift in running toward an earlier period in the dark phase remains to be determined. It is generally accepted that alterations in immune status result in behavioral changes [29]. To et al. reported that the plasma tumor necrosis factor-α concentration was higher in the light phase and the later periods of the dark phase in mice with CIA [30]. Therefore, the earlier period in the dark phase, during which most wheel running was observed in mice with CIA in the present study, corresponds well to the period with the lowest tumor necrosis factor-α concentration in their study. Therefore, it is likely that circadian phase-dependent changes in the immune system underlie such phasic changes in voluntary behavior in RA models. This hypothesis needs to be confirmed directly in future studies.

### Temperature preference test

In mammals, the environmental temperature is detected by a series of transient receptor potential channels activated in overlapping narrow ranges of specific temperatures ranging from about 17 to 43 °C [31]. Detection of temperature using these transient receptor potential channels has advantages in animals, including homeostatic optimization of metabolism for conservation of body temperature and detection of contact with a substance that is hot enough to damage tissues. The most interesting and novel finding of this study is that the CIA group, but not the CFA group or the saline group, preferred middle-range floor temperature of 31–43 °C to one of 25 °C. This tendency was clearly observed after day 28 during our observation period (Fig. 4b). This finding clearly indicates that the temperature-comfort relationship is altered in mice with CIA. This interpretation is supported by the longer dwell time and decreased transition between the plates in phases I and II, indicating that the mice preferred to stay on the warmer 31–43 °C plate without frequently walking off it (Fig. 4b, Additional file 5: Figure S5B). This change in temperature-comfort relationship is reminiscent of the preference of human patients with RA for warm baths (36–37 °C), which relieve pain and provide a basis for conventional spa therapy [32, 33]. It is thus speculated that the increased preference for a warm temperature observed in the CIA mice in this study might reflect the subjective “feeling” of animals that a warmer temperature can reduce nociceptive sensation. The neural mechanism underlying this change is unknown. Given that thermoregulatory behavior is mediated by neuronal activity in the lateral parabrachial nucleus [34], which is also a major target of nociceptive neurons in the superficial layers of the dorsal horn [35], it would be interesting to investigate the role of this nucleus in temperature preference behavior and its functional significance in terms of inflammation [36] and energy expenditure in mice with CIA.

It has been shown that sensitivity to the noxious thermal stimulation is increased in the RA models [7, 19, 37, 38], indicating that the nocifensive function of thermosensation (i.e., detection of contact with a noxious high temperature) is potentiated in mice with CIA. In this study, the plate with the temperature ranging from 43 to 49 °C (phase III in Fig. 4 and Additional file 5: Figure S5) was consistently avoided by the saline, CFA, and CIA groups, indicating that this temperature range would provoke aversive sensation in mice receiving any of these treatments. The mice in the present experiments could rapidly escape from an aversive environment, so it is suggested that the ability of mice to detect a potentially noxious thermal sensation and avoid the tissue damage that would occur at a temperature higher than 50 °C is essentially unaltered in mice with CIA.

Interestingly, in phases I and II after day 28, the dwell time was significantly longer and the transition number was significantly smaller in CIA groups than the other groups (Additional file 5: Figure S5B). Considering the preference of CIA mice to the warmer-temperature plate in these phases (Fig. 4a, b), this result means that the CIA mice not only chosen the warmer temperature plate more frequently but also stayed in this preferred-temperature plate with less frequent moves from one to another compared with other groups once they entered this plate. This decreased plate changes might be attributed either to their reluctance to walk between the plates presumably because of the joint discomfort/pain and/or to their stronger adherence to the preferred environment in the CIA group. This interpretation is also consistent with the reduced rotation number in the wheel running test during this phase. In contrast, in phase III, where the plate temperature was above 43 °C at the higher-temperature plate, mice stayed for longer time in the 25 °C plate and moved much less frequently between plates regardless of the group. One plausible interpretation is that this higher temperature is consistently aversive and it surpasses the distinct temperature preference of each group. Altogether, this temperature preference test can evaluate, in addition to the specific temperature preference of the disease model, more integrated strategy against potentially harmful events rather than the changes in evoked nociception threshold. It is concluded that despite the alterations in the temperature preference in non-aversive environments, the CIA mice maintain behavioral strategy against potential risk.

### Discordance of correlation between arthritis score and voluntary behavior

As discussed above, the voluntary wheel running test and temperature preference test can evaluate voluntary choice of behavior in animals. Another important and novel finding of this report is that these measures of voluntary behavior became dissociated from the objective evaluation of symptoms of arthritis in the course of treatment with an anti-rheumatic drug (tofacitinib; Figs. 3f and 5f). This gap between behavior and symptoms has not been reported before in an animal model of RA. This gap became manifest on day 38, 17 days after the booster administration in the presence and absence of tofacitinib, which was continuously administered from day 16 using implanted sustained-release pump. Before and after day 38, the wheel running and temperature preference were negatively and positively, respectively, correlated with the arthritis symptom. As the arthritis score remained significantly smaller in the tofacitinib group than in the vehicle group after day 31, the transient loss of this significant correlation around day 38 suggests persisting aberrance of sensory-associated behavior despite earlier subsidence of the inflammation in a subset of animals. This discrepancy is a reminiscence of a similar discordance between subjective complaints of pain and objective symptoms of arthritis which is a frequently observed issue in clinical practice. For example, Studenic et al. reported that RA activity evaluated by patient’s global assessment was discordant with that evaluated by physicians mostly because of the mismatch between pain and joint swelling evaluations [1]. These “patient-physician gaps” when evaluating disease activity can be found in more than half of patients with RA [3], even after treatment with anti-tumor necrosis factor agents [39], so are problematic in RA medicine because they reduce the likelihood of remission [4, 40, 41]. One of the plausible interpretations for the reduced arthritis-locomotion correlation on day 38 would be that the time course of the resolution of pain and associated behavior is distinct from that for the symptoms of arthritis, presumably due to the difference in their pathophysiological backgrounds.

### Limitations in evaluating pain

Pain is subjective [6]. Reasonable and objective evaluation of “pain” in animal models [42] as well as in patients without subjective expression [43] has long been a matter of debate. The lack of proper evaluation of pain in animals, not the nocifensive reflexogenic behavior, has been an obstacle to the development of novel pharmacotherapies against spontaneous long-lasting pain in human patients [44]. In this study, we primarily interpreted the decreased spontaneous wheel running in arthritic animals as signs of pain because (1) similar types of RA models show significantly decreased threshold to mechanical touch in the same clinical period as decreased wheel running in this study [7, 38, 45] and (2) the model rats of neuropathic pain show a similar decrease in wheel running immediately following the establishment of neuropathic pain symptom [46]. While it is possible that the altered behavior is an expression of pain, one cannot exclude other factors, such as destruction of the joint [7], psychological distress and fatigue [5], and sickness response, which underlie the decreased wheel running, as stated above. The preference of mice with CIA to warm environment found in this study is not directly associated with pain, but it is possible that activation of the peripheral nerves sensitive to these temperature ranges would modify the central integration and preference/avoidance pattern of environment temperature [47]. It is important to note that the conclusions of this study are confirmed only in male mice. As distinct mechanisms underlie the establishment of pain-associated behavior [48], it is an interesting future subject to study the sex-dependent difference in the behavior of the mice with CIA.

Recent advances in pain neuroscience have demonstrated that long-lasting pain causes neuroplastic changes in the central nervous system [49], changes in synaptic transmission [50], and changes in the morphofunctional properties of glial cells [7, 51, 52]. Our study is the first to demonstrate that not only the joints, but also the central glial systems, are affected in mice with CIA and that the latter remain activated in the spinal cord even after recovery of the arthritis score by tofacitinib (Additional file 1: Figure S1E–G). It is very likely that this distinct difference in the time course of recovery between symptoms of arthritis and activation of the central nervous system in the course of drug therapy would underlie the objective-subjective gap in the treatment of patients with RA.

## Conclusions

We described for the first time significant alterations of the voluntary behavior of the mice with CIA during the clinical periods, indicating that the overall physical/motivational states and its circadian variation, as well as the specific preference to a certain environmental temperature, are modified in the mice with CIA, as observed in human patients. Some of these did not parallel with the conventional arthritis scores, particularly during the pharmacotherapy suggesting that mice with CIA show not only the peripheral symptoms but also central consequences. The use of these approaches would also help clarify the biological mechanisms underlying physician-patient discordance in the assessment of RA.

## Supplementary information

**Additional file 1: Figure S1.** (A–C) Time course of the arthritis score and incidence of arthritis (A), change in average hind paw thickness (B), and change in body weight (C) in mice injected with saline, CFA, or bovine type II collagen (CIA). According to the change in arthritis score (squares) and incidence of arthritis (triangles), the observation period was divided into pre-clinical (days 18–25), early clinical (days 26–34), and late clinical (days 35–42; A, see text for details). The values are shown as the mean ± standard error of the mean. *p < 0.05, saline vs CIA; ^#^p < 0.05, CFA vs CIA, by one-way ANOVA followed by Tukey’s test (B, C). (D) Hematoxylin and eosin staining of the ankle joint on day 42 in the saline, CFA, and CIA groups. Scale bar = 100 μm. (E) Immunohistochemistry of GFAP at the dorsal horn of L4, which receives sensory afferents from the ankle joint, on day 42 in representative mice from the saline, CFA, and CIA groups. The insets in the top right corner are magnified versions of the region indicated with the white square. The dotted line indicates the border between the white and gray matter. L, lateral side; D, dorsal side. Scale bars = 50 μm. (F) mRNA expression of *Gfap* and *Cd11b* in the spinal cord at L3–L5 on day 45 in the saline, CFA, and CIA groups. The values are presented relative to the average value in the saline group. The bars indicate average expression and each open circle indicates a value from one mouse. The numbers of mice are indicated in the bars. *p < 0.05, saline vs CIA; ^#^p < 0.05, CFA vs CIA, n.s., not significant by one-way ANOVA followed by Tukey’s test. (G) mRNA expression of *Gfap* in the spinal cord at L3–L5 on day 45 in vehicle-treated and tofacitinib-treated CIA mice. The values are shown relative to the average in vehicle-treated CIA mice. The bars indicate average expression and each circle indicates a value from one mouse. The numbers of mice are indicated in bars. No significant difference between vehicle vs tofacitinib, Student’s *t*-test.

**Additional file 2: Figure S2.** Representative examples of the time course of number of rotations and arthritis scores in each group. Color scale plots: abscissa, time of day (24-h system; 7 am to 7 pm, light phase; 7 pm to 7 am, dark phase); ordinate, day after first injection. Color code for number of rotations is shown on the right. The white color represents the period during which recording was stopped for assessment of arthritis and replacement of cages. RF, LF, RH, and LH indicate the arthritis score for the right forepaw, left forepaw, right hind paw, and left hind paw, respectively. The arthritis scale score (0–3) is shown at the right. The histograms below each plot represent the time course of number of rotations on day 23 (pale color) and day 34 (dark color). Centroid time is shown with arrowheads (day 23, pale color; day 34, dark color). CFA, complete Freund’s adjuvant; CIA, collagen-induced arthritis.

**Additional file 3: Figure S3.** (A) Mean number of rotations per hour in the pre-clinical period (days 18–25) in the light phase (7 am to 7 pm) and dark phase (7 pm to 7 am) in the saline, CFA, and CIA groups. The bars indicate the average number of rotations and each open circle indicates a value for a single mouse. L, light phase; D, dark phase. ^††^*p* < 0.01, by paired *t*-test; n.s., no statistically significant difference between the three groups by one-way ANOVA followed by Tukey’s test. (B) Time course of the arthritis score in the saline, CFA, and CIA groups in experiment 1. The values are shown as the mean ± standard error of the mean. (C) Time course of centroid time in the saline, CFA, and CIA groups. The values are shown as the mean ± standard error of the mean. (D) The mean centroid time in the pre-clinical, early clinical, and late clinical periods in the saline, CFA, and CIA groups. Each open diamond indicates a value from a single mouse. The horizontal bar indicates the average centroid time in each group. *p < 0.05, saline vs CIA; ^#^p < 0.05, CFA vs CIA, by one-way ANOVA followed by Tukey’s test. (E) Time course of centroid time in tofacitinib-treated and vehicle-treated mice with CIA. (F) Mean centroid time in the pre-clinical, early clinical, and late clinical periods in the vehicle-treated and tofacitinib-treated mice. Each diamond indicates a value from one mouse. The horizontal bar indicates the average centroid time. *p < 0.05, vehicle vs tofacitinib, n.s., not statistically significant by the Student’s *t*-test. (A–D) and (E, F) relate to experiment 1 and experiment 2, respectively.

**Additional file 4: Figure S4.** Representative examples of floor temperature-dependent spontaneous displacement of mice in a two-floored area with distinct temperatures on day 38. The photograph on the left shows a frame of a video image captured by a CCD camera under red illumination, which was invisible to the mice. The white dot indicates the center of gravity of the body of the mouse being tested. The graphs above indicate the y axis position of the mouse (abscissa) during 24 min of observation (ordinate). The graphs below show the time-dependent changes in preference ratio calculated every 2 min for this mouse. The temperature of plate 1 was increased from 25 °C to 49 °C for 24 min (1 °C/min; see color scale, top right) while maintaining the plate 2 temperature at 25 °C throughout. Note that the CIA mouse preferred to stay in the temperature-augmenting plate in phase II and mice in all the study groups avoided remaining longer on the hotter plate in phase III.

**Additional file 5: Figure S5.** (A) Time-dependent changes in dwell time on plate 1 and transition number between the two plates with fixed (25 °C) and increasing temperature (1 °C/min from 25 °C for 24 min) in the pre-clinical period (days 19–24), early clinical period (days 28–33) and late clinical period (days 38–42) in the saline, CFA, and CIA groups. The ordinate indicates the dwell time (upper) and transition number (lower) per 2 min. The color scale above indicates the temperature of the plates at each time point. The values are shown as the mean ± standard error of the mean. *p < 0.05, saline vs CIA; #p < 0.05, CFA vs CIA, Kruskal-Wallis test followed by the Steel-Dwass post hoc multiple comparison test for dwell time and one-way ANOVA followed by Tukey’s test for transition number. (B) Time course of dwell time (upper) and transition number (lower) per 6 min in each phase in the saline, CFA, and CIA groups. Values are shown as the mean ± standard error of the mean. *p < 0.05, saline vs CIA; ^#^p < 0.05, CFA vs CIA, Kruskal-Wallis test followed by the Steel-Dwass post hoc multiple comparison test for dwell time and by one-way ANOVA followed by Tukey’s test for transition number. (C) Test time-dependent choice of 2 plates on day 42 in tofacitinib-treated and vehicle-treated mice with CIA. The ordinate indicates the dwell time (upper) and transition number (lower). The color scale above indicates the temperature of the plates at each time point. The values are shown as the mean ± standard error of the mean. *p < 0.05, **p < 0.01, vehicle vs tofacitinib, Mann-Whitney *U* test for dwell time and the Student’s *t*-test for transition number.

## Data Availability

The datasets used and analyzed during the current study are available from the corresponding author on reasonable request.

## Abbreviations

ANOVA: Analysis of variance
CFA: Complete Freund’s adjuvant
CIA: Collagen-induced arthritis
GFAP: Glial fibrillary acidic protein
JAK: Janus kinase
PEG: Polyethylene glycol
RA: Rheumatoid arthritis
