# Supplementary figures and images for: Alterations of voluntary behavior in the course of disease progress and pharmacotherapy in mice with collagen-induced arthritis
Source: Arthritis Res Ther. 2019 Dec 12;21:284. doi: 10.1186/s13075-019-2071-z (PMC6909634; doi:10.1186/s13075-019-2071-z)

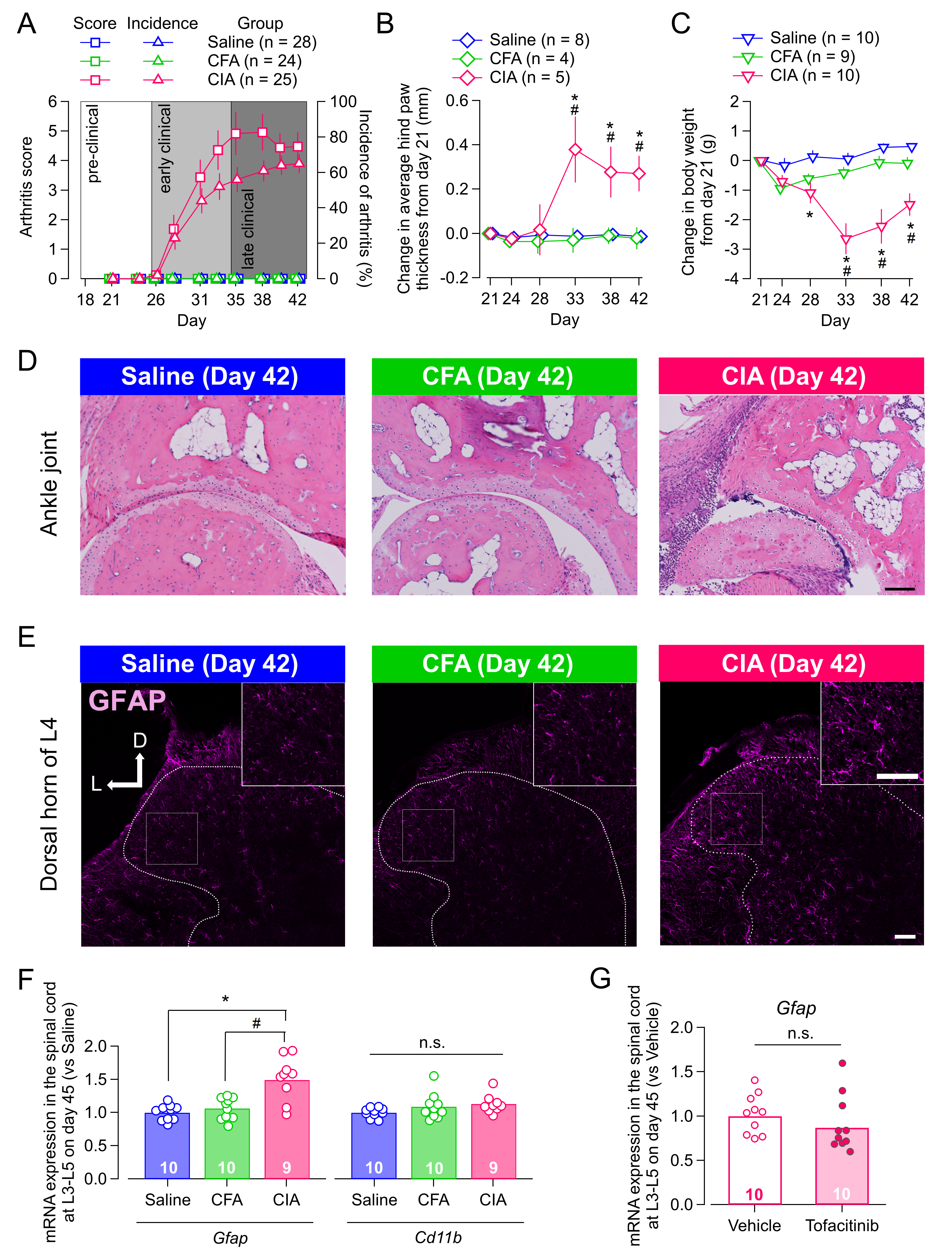

Supplement: Supplementary file 1 — Additional file 1: Figure S1. (A–C) Time course of the arthritis score and incidence of arthritis (A), change in average hind paw thickness (B), and change in body weight (C) in mice injected with saline, CFA, or bovine type II collagen (CIA). According to the change in arthritis score (squares) and incidence of arthritis (triangles), the observation period was divided into pre-clinical (days 18–25), early clinical (days 26–34), and late clinical (days 35–42; A, see text for details). The values are shown as the mean ± standard error of the mean. *p < 0.05, saline vs CIA; #p < 0.05, CFA vs CIA, by one-way ANOVA followed by Tukey’s test (B, C). (D) Hematoxylin and eosin staining of the ankle joint on day 42 in the saline, CFA, and CIA groups. Scale bar = 100 μm. (E) Immunohistochemistry of GFAP at the dorsal horn of L4, which receives sensory afferents from the ankle joint, on day 42 in representative mice from the saline, CFA, and CIA groups. The insets in the top right corner are magnified versions of the region indicated with the white square. The dotted line indicates the border between the white and gray matter. L, lateral side; D, dorsal side. Scale bars = 50 μm. (F) mRNA expression of Gfap and Cd11b in the spinal cord at L3–L5 on day 45 in the saline, CFA, and CIA groups. The values are presented relative to the average value in the saline group. The bars indicate average expression and each open circle indicates a value from one mouse. The numbers of mice are indicated in the bars. *p < 0.05, saline vs CIA; #p < 0.05, CFA vs CIA, n.s., not significant by one-way ANOVA followed by Tukey’s test. (G) mRNA expression of Gfap in the spinal cord at L3–L5 on day 45 in vehicle-treated and tofacitinib-treated CIA mice. The values are shown relative to the average in vehicle-treated CIA mice. The bars indicate average expression and each circle indicates a value from one mouse. The numbers of mice are indicated in bars. No significant difference between vehicle vs tofa [file 13075_2019_2071_MOESM1_ESM.png]

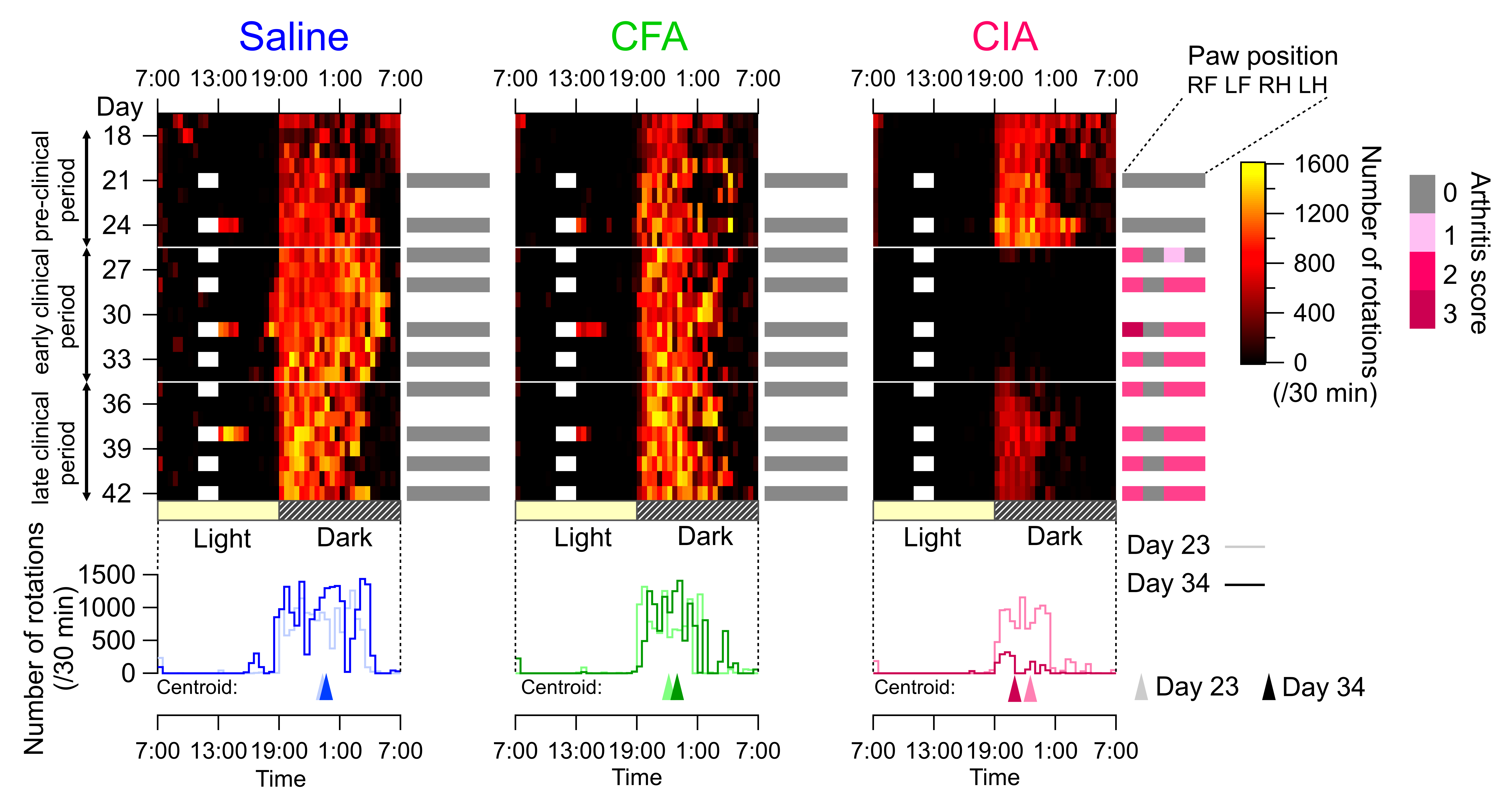

Supplement: Supplementary file 2 — Additional file 2: Figure S2. Representative examples of the time course of number of rotations and arthritis scores in each group. Color scale plots: abscissa, time of day (24-h system; 7 am to 7 pm, light phase; 7 pm to 7 am, dark phase); ordinate, day after first injection. Color code for number of rotations is shown on the right. The white color represents the period during which recording was stopped for assessment of arthritis and replacement of cages. RF, LF, RH, and LH indicate the arthritis score for the right forepaw, left forepaw, right hind paw, and left hind paw, respectively. The arthritis scale score (0–3) is shown at the right. The histograms below each plot represent the time course of number of rotations on day 23 (pale color) and day 34 (dark color). Centroid time is shown with arrowheads (day 23, pale color; day 34, dark color). CFA, complete Freund’s adjuvant; CIA, collagen-induced arthritis. [file 13075_2019_2071_MOESM2_ESM.png]

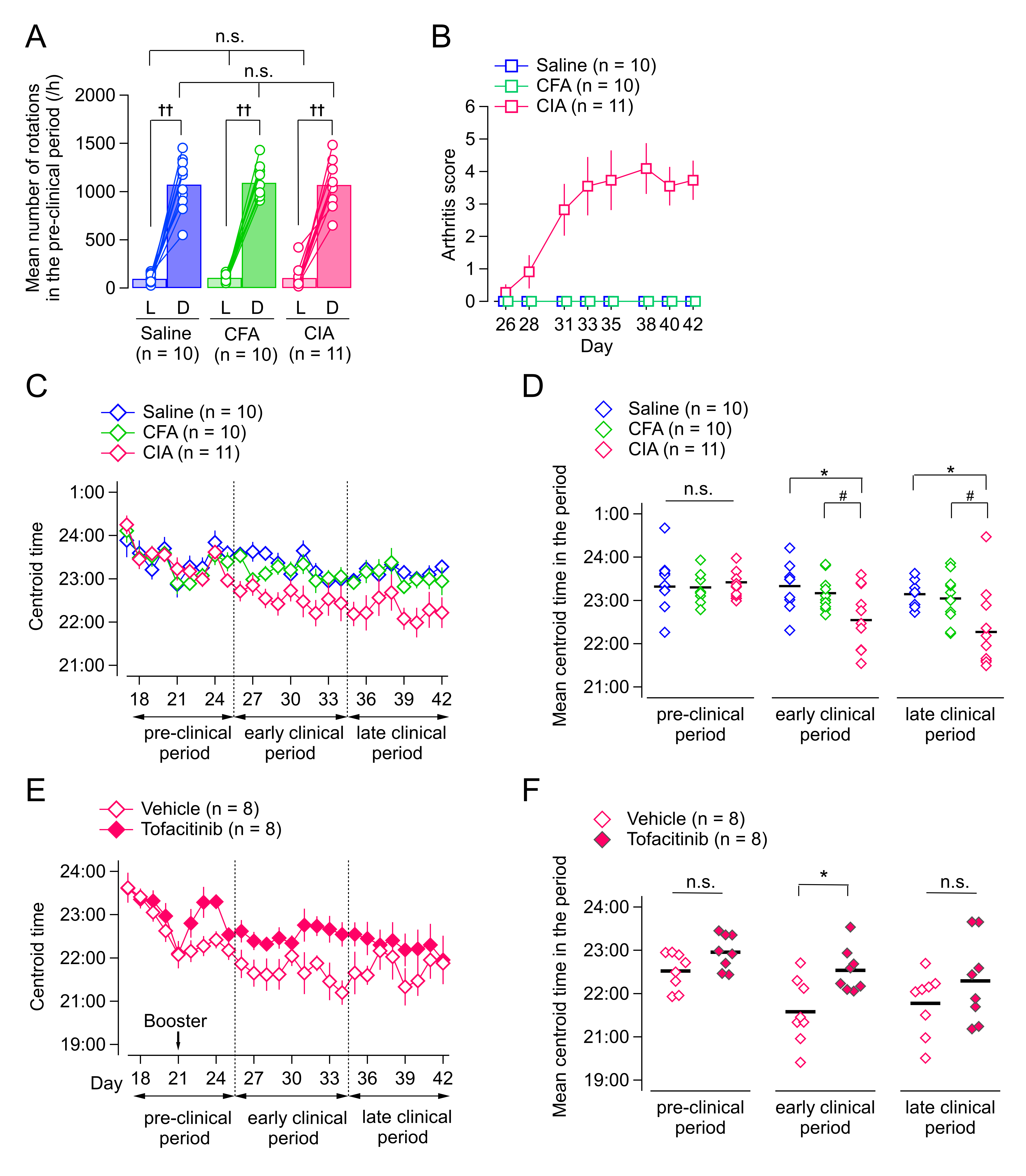

Supplement: Supplementary file 3 — Additional file 3: Figure S3. (A) Mean number of rotations per hour in the pre-clinical period (days 18–25) in the light phase (7 am to 7 pm) and dark phase (7 pm to 7 am) in the saline, CFA, and CIA groups. The bars indicate the average number of rotations and each open circle indicates a value for a single mouse. L, light phase; D, dark phase. ††p < 0.01, by paired t-test; n.s., no statistically significant difference between the three groups by one-way ANOVA followed by Tukey’s test. (B) Time course of the arthritis score in the saline, CFA, and CIA groups in experiment 1. The values are shown as the mean ± standard error of the mean. (C) Time course of centroid time in the saline, CFA, and CIA groups. The values are shown as the mean ± standard error of the mean. (D) The mean centroid time in the pre-clinical, early clinical, and late clinical periods in the saline, CFA, and CIA groups. Each open diamond indicates a value from a single mouse. The horizontal bar indicates the average centroid time in each group. *p < 0.05, saline vs CIA; #p < 0.05, CFA vs CIA, by one-way ANOVA followed by Tukey’s test. (E) Time course of centroid time in tofacitinib-treated and vehicle-treated mice with CIA. (F) Mean centroid time in the pre-clinical, early clinical, and late clinical periods in the vehicle-treated and tofacitinib-treated mice. Each diamond indicates a value from one mouse. The horizontal bar indicates the average centroid time. *p < 0.05, vehicle vs tofacitinib, n.s., not statistically significant by the Student’s t-test. (A–D) and (E, F) relate to experiment 1 and experiment 2, respectively. [file 13075_2019_2071_MOESM3_ESM.png]

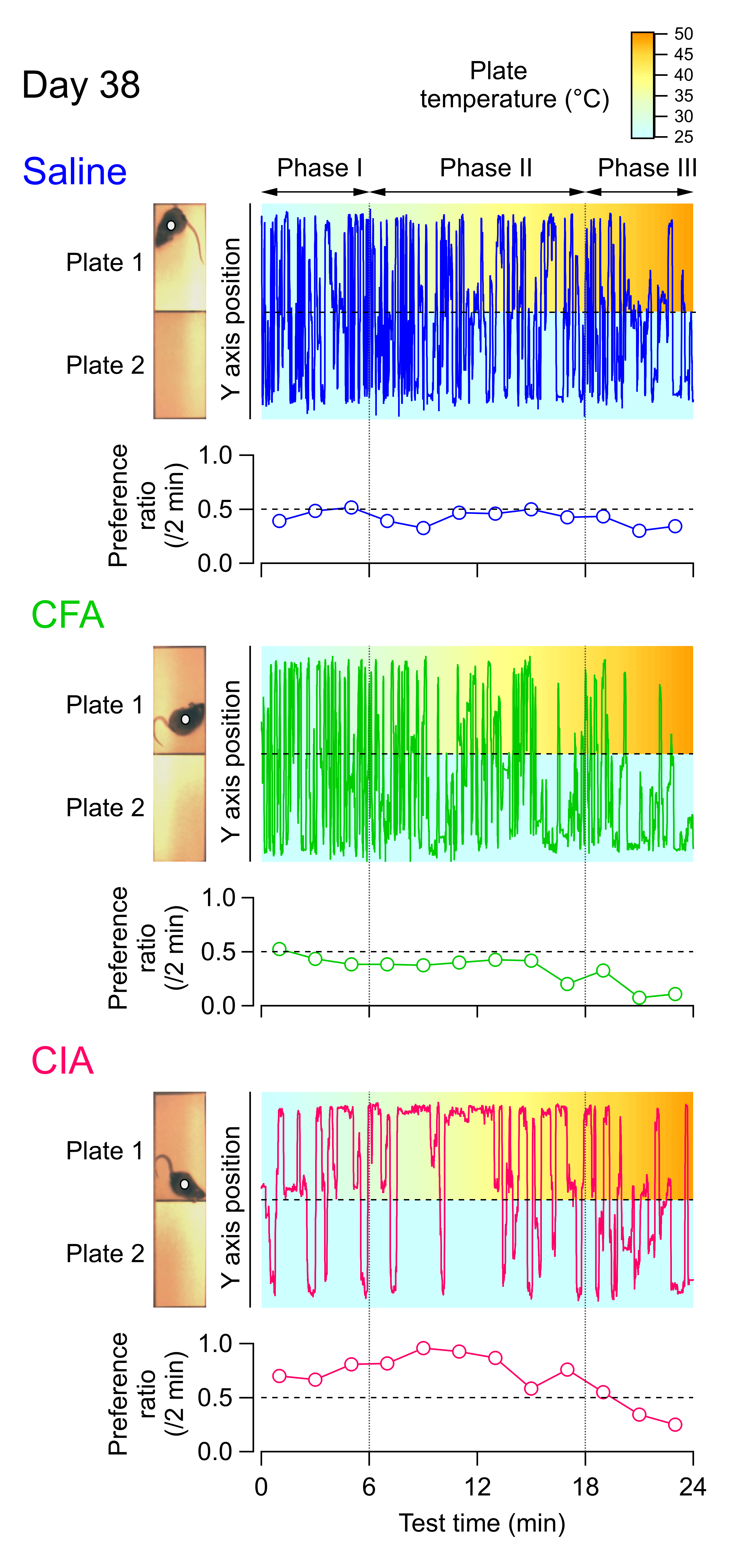

Supplement: Supplementary file 4 — Additional file 4: Figure S4. Representative examples of floor temperature-dependent spontaneous displacement of mice in a two-floored area with distinct temperatures on day 38. The photograph on the left shows a frame of a video image captured by a CCD camera under red illumination, which was invisible to the mice. The white dot indicates the center of gravity of the body of the mouse being tested. The graphs above indicate the y axis position of the mouse (abscissa) during 24 min of observation (ordinate). The graphs below show the time-dependent changes in preference ratio calculated every 2 min for this mouse. The temperature of plate 1 was increased from 25 °C to 49 °C for 24 min (1 °C/min; see color scale, top right) while maintaining the plate 2 temperature at 25 °C throughout. Note that the CIA mouse preferred to stay in the temperature-augmenting plate in phase II and mice in all the study groups avoided remaining longer on the hotter plate in phase III. [file 13075_2019_2071_MOESM4_ESM.png]

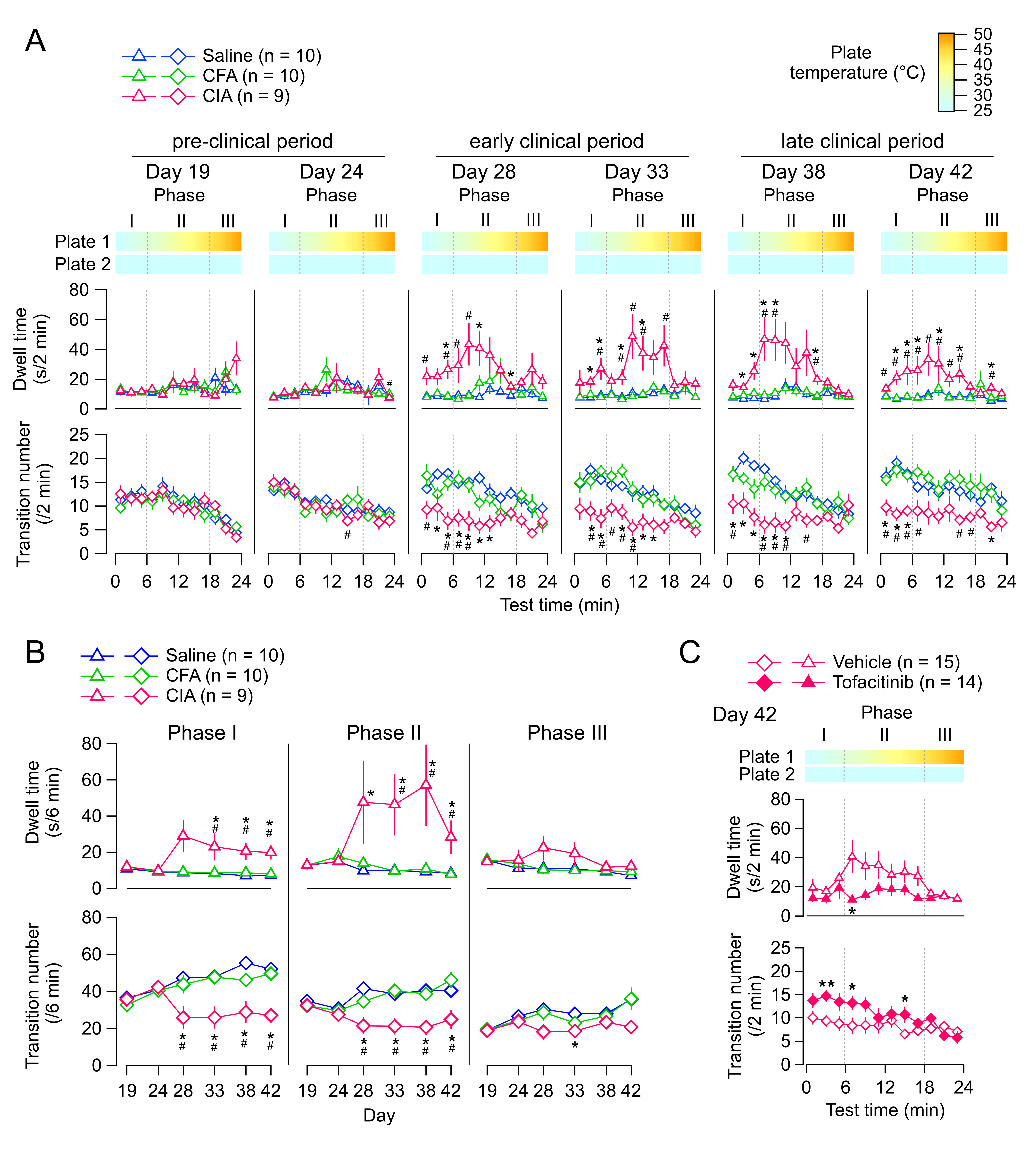

Supplement: Supplementary file 5 — Additional file 5: Figure S5. (A) Time-dependent changes in dwell time on plate 1 and transition number between the two plates with fixed (25 °C) and increasing temperature (1 °C/min from 25 °C for 24 min) in the pre-clinical period (days 19–24), early clinical period (days 28–33) and late clinical period (days 38–42) in the saline, CFA, and CIA groups. The ordinate indicates the dwell time (upper) and transition number (lower) per 2 min. The color scale above indicates the temperature of the plates at each time point. The values are shown as the mean ± standard error of the mean. *p < 0.05, saline vs CIA; #p < 0.05, CFA vs CIA, Kruskal-Wallis test followed by the Steel-Dwass post hoc multiple comparison test for dwell time and one-way ANOVA followed by Tukey’s test for transition number. (B) Time course of dwell time (upper) and transition number (lower) per 6 min in each phase in the saline, CFA, and CIA groups. Values are shown as the mean ± standard error of the mean. *p < 0.05, saline vs CIA; #p < 0.05, CFA vs CIA, Kruskal-Wallis test followed by the Steel-Dwass post hoc multiple comparison test for dwell time and by one-way ANOVA followed by Tukey’s test for transition number. (C) Test time-dependent choice of 2 plates on day 42 in tofacitinib-treated and vehicle-treated mice with CIA. The ordinate indicates the dwell time (upper) and transition number (lower). The color scale above indicates the temperature of the plates at each time point. The values are shown as the mean ± standard error of the mean. *p < 0.05, **p < 0.01, vehicle vs tofacitinib, Mann-Whitney U test for dwell time and the Student’s t-test for transition number. [file 13075_2019_2071_MOESM5_ESM.png]
